# Secondary Sites of the C-type Lectin-Like Fold

**DOI:** 10.1002/chem.202400660

**Published:** 2024-04-30

**Authors:** Jonathan Lefèbre, Torben Falk, Yunzhan Ning, Christoph Rademacher

**Affiliations:** [a] J. Lefèbre, T. Falk, Y. Ning, C. Rademacher Department of Pharmaceutical Sciences, University of Vienna, Vienna, Austria; [b] J. Lefèbre, T. Falk, Y. Ning Vienna Doctoral School of Pharmaceutical, Nutritional and Sport Sciences, University of Vienna, Vienna, Austria; [c] J. Lefèbre, T. Falk, Y. Ning, C. Rademacher Department of Microbiology, Immunology and Genetics, University of Vienna, Max F. Perutz Labs, Vienna, Austria

## Abstract

C-type lectins are a large superfamily of proteins involved in a multitude of biological processes. In particular, their involvement in immunity and homeostasis has rendered them attractive targets for diverse therapeutic interventions. They share a characteristic C-type lectin-like domain whose adaptability enables them to bind a broad spectrum of ligands beyond the originally defined canonical Ca^2+^-dependent carbohydrate binding. Together with variable domain architecture and high-level conformational plasticity, this enables C-type lectins to meet diverse functional demands. Secondary sites provide another layer of regulation and are often intricately linked to functional diversity. Located remote from the canonical primary binding site, secondary sites can accommodate ligands with other physicochemical properties and alter protein dynamics, thus enhancing selectivity and enabling fine-tuning of the biological response. In this review, we outline the structural determinants allowing C-type lectins to perform a large variety of tasks and to accommodate the ligands associated with it. Using the six well-characterized Ca^2+^-dependent and Ca^2+^-independent C-type lectin receptors DC-SIGN, langerin, MGL, dectin-1, CLEC-2 and NKG2D as examples, we focus on the characteristics of non-canonical interactions and secondary sites and their potential use in drug discovery endeavors.

## Graphical abstract

**Figure:**
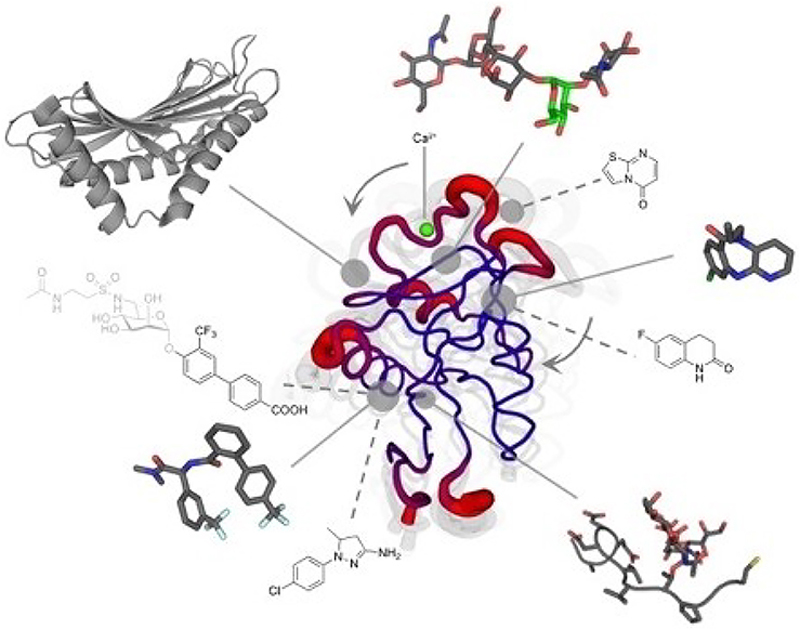
C-type lectins, a large superfamily of proteins, are crucial in immunity and homeostasis. Their C-type lectin-like domain enables them to bind a wide range of ligands, including those not dependent on Ca^2+^ interactions. This adaptability is further enhanced by their variable domain architecture and conformational plasticity. Secondary binding sites, often located away from the primary site, contribute to the C-type lectin functional diversity by accommodating different ligands, thus offering potential for drug discovery by fine-tuning biological responses and increasing selectivity.

## Introduction

Protein-carbohydrate interactions play a fundamental role in multicellular systems, encompassing many processes ranging from immunity and cell-cell communication to modulation of cellular growth and development.^[1]^ Within this complex interplay, carbohydrate binding proteins, also referred to as lectins, are specifically tuned to recognize and decode glycan structures of varying composition and stereochemistry. In mammals, the largest family of lectins are the C-type lectins (CTLs) – a diverse protein superfamily unified by the presence of a characteristic C-type lectin-like domain (CTLD).^[2]^ Originally, the CTLD has been described to bind glycans in a Ca^2+^-dependent manner and is often referred to as carbohydrate recognition domain (CRD) for glycan binding CTLs. Nevertheless, the CTLD can tolerate substantial modifications to interact with glycans, proteins, and lipids in the absence of Ca^2+^-coordination, while maintaining a robust fold.^[2–4]^ Combined with varying overall domain architectures and topology, this renders CTLs highly adaptable to the structural requirements of a multitude of functions, including discrimination between self and non-self, cellular adhesion, endocytic uptake, and modulation of intracellular signaling pathways.^[5–9]^

To date, more than 700 CTL-encoding sequences have been identified across taxa and led to the definition of at least 16 groups based on structural and phylogenetic relationships.^[2]^ In humans and other higher vertebrates, transmembrane CTL receptors (CLRs) belonging to groups II (Asialogylcoprotein receptor (ASGPR) & dendritic cell (DC) receptors), IV (Selectins), V (Natural killer (NK) cell receptors) and VI (multi-CTLD endocytic receptors) that directly or indirectly shape homeostasis and immunological response to infection and cancer have gained particular attention.^[9–11]^ Catalyzed by their biomedical importance, an increasing amount of structural and functional information for these receptors is now available, contributing to our understanding of their biology and revealing new opportunities to interfere with it for therapeutic intervention.

Reminiscent of their structural variability, single well-studied examples document how conformational plasticity modulates CLR function.^[12]^ Ranging from subtle changes in loop mobilities to larger rearrangements of the CLR domain architecture, intrinsic and ligand-induced dynamics can affect, for example, Ca^2+^ and ligand binding affinity related to endocytic uptake and cellular adhesion.^[6,13–20]^ Yet, despite considerable progress, a generalized mechanistic understanding of the structural requirements translating ligand binding at the CTLD into cellular response, such as signaling cascades and cargo routing, remains elusive.^[21]^ For instance, for group II CTL Dendritic cell-specific ICAM-3-grabbing non-integrin (DC-SIGN), differential signaling pathways leading to activation or suppression of proinflammatory response upon binding to fucose or mannose-containing ligands have been reported, respectively.^[22,23]^ While conformational changes were suggested to be the origin of this contrasting activity, neither X-ray crystallographic structures nor nuclear magnetic resonance (NMR) indicated significant differences between mannose or fucose bound and unbound DC-SIGN CTLD.^[24–26]^

To achieve precise functional regulation, many proteins make use of secondary sites remote from the primary binding site.^[27]^ Ligand binding at these sites may have improved selectivity and can induce conformational changes that directly impact protein function, such as intracellular signaling, or indirectly *via* modulation of the primary binding site and co-factor binding sites.^[28,29]^ Considering the promiscuity of the CTLD to interact with diverse ligand chemistries and that only a minority of CTLs possess motifs for canonical Ca^2+^-dependent carbohydrate binding, the availability of functional or druggable secondary sites in CTLs is likely and has been documented for individual cases.^[12,30]^

In the following sections, we provide an overview of current knowledge on the domain architecture, ligand binding characteristics, and conformational plasticity of CTLs. Due to the availability of structural and functional data, we focus on the biomedical important CLRs of groups II, IV, V, and VI and highlight six well-documented example receptors, showcasing the role of secondary sites and non-canonical interactions in biological function as well as for drug discovery purposes.

### Domain Architecture

CLRs of groups II, IV, V, and VI are single-pass transmembrane proteins that contain an extracellular domain comprising at least one CTLD and a stalk region that connects to a transmembrane domain and a cytoplasmic domain.^[31]^ While the CTLD fold is conserved, subtle changes to its structure and differences in the extracellular domain separate the groups and members of each.

CTL group II is a large group of type II transmembrane proteins that comprises some of the best characterized CLRs. Their extracellular domain contains a single Ca^2+^-dependent carbohydrate binding CTLD and a variable stalk region followed by a transmembrane domain and a short cytoplasmic tail that can contain signaling and sorting motifs.^[3,32]^ For prominent examples of this group, such as DC-SIGN, langerin, and macrophage galactose lectin (MGL), an elongated stalk domain enables homooligomerization *via* coiled-coil interactions and facilitates multivalent recognition of glycosylated ligands.^[33–35]^

For other members of group II, such as macrophage inducible C-type lectin (mincle) and macrophage C-type lectin (MCL), heterodimerization *via* the stalk and an intermolecular disulfide bond impacts expression, endocytosis and signaling activity.^[36,37]^ CTLs of group V display similar domain architecture and have been separated from group II based on phylogenetic analysis.^[3]^ Their CTLD lacks Ca^2+^-coordination motifs, thus also canonical Ca^2+^-dependent carbohydrate binding activity. Instead, group V CLRs such as Lectin-like oxidized low-density lipoprotein (oxLDL) receptor-1 (LOX-1], interact with a broader spectrum of ligands with binding sites often located remote from the canonical carbohydrate binding site (CBS) found in other CLRs.^[38–41]^ Groups IV and VI are smaller, less heterogenous groups with more complex domain architecture compared to groups II and V. Both are type I transmembrane proteins containing a cassette-like arrangement of additional domains within their extracellular domain. Selectins of group IV share a single Ca^2+^-dependent carbohydrate binding CTLD, an epidermal growth factor (EGF)-like domain, and a variable number of consensuses repeat (CR) domains. Group VI CLRs, such as DEC-205 and mannose receptor (MR), have an additional ricin-like and a fibronectin type 2 domain, which are followed by eight to ten non-equal CTLDs with limited sequence identity and varying or no Ca^2+^ and glycan-binding activity.^[11,20,42,43]^

Contrasting the extracellular domain architecture, the cytoplasmic domain and its associated signaling motifs and adaptor proteins are generally CTL group independent. They have been broadly categorized based on the presence of immunoreceptor tyrosine-based activating or inhibiting motifs (ITAM, hemITAM, ITIM), association with ITAM-containing adaptor proteins (ITAM-coupled) as well as, ITAM-ITIM-independent signaling activity.^[32]^ The functions of these motifs are of high interest and have been reviewed extensively elsewhere.^[7,10]^

### C-type Lectin-Like Domain Fold

**Figure 1:**
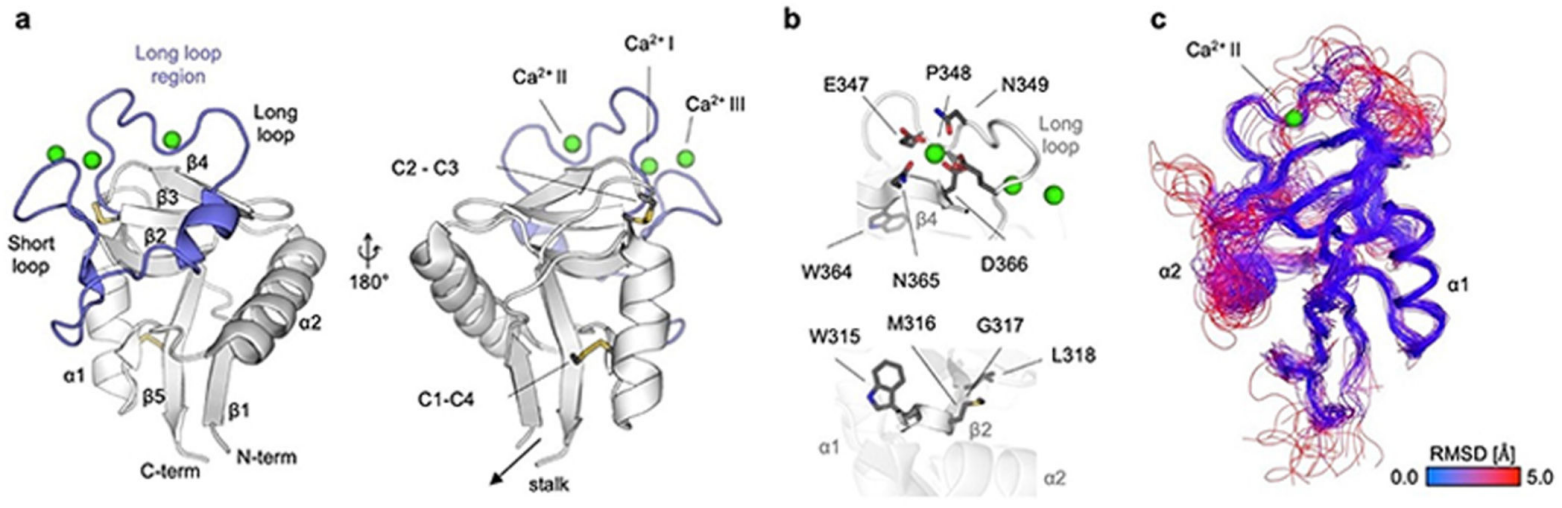
Structure of the C-type lectin-like domain (CTLD). (a) The C-type lectin-like fold with secondary structure element numbering using DC-SIGN (PDB ID: 1SL4) as an example. The long loop region is highlighted in blue. Disulfide bonds are shown as sticks. Ca^2+^ ions are shown in green spheres. The fourth Ca^2+^ site located between α2, β1, and β5 is not shown. Secondary structure element numbering according to Zelensky and Gready.^[3]^ (b) The EPN and WND motifs (top) in the long loop region and β4, respectively, coordinate the Ca^2+^ ion in Ca^2+^ site II. The WIGL (WMGL in DC-SIGN) motif (bottom) in β2 forms the hydrophobic core. DC-SIGN (PDB ID: 1SL4) is shown as an example CTLD. (c) Structural alignment of 38 unique PDB IDs of human CTLDs. While the CTLD fold is structurally highly conserved, the long loop region, harboring the Ca^2+^ sites and the canonical CBS, are structurally diverse. Coloring of the structures according to RMSD between X-ray crystallographic structures.

The CTLD is a compact globular fold of 110–140 residues that can be described as a double-looped structure with two antiparallel β sheets and two α helices (Figure 1a).^[3]^ The overall domain is stabilized by at least two highly conserved intrachain disulfide bridges. Additional interchain disulfide bridges have been proposed to facilitate homo- and heterodimer formation, especially in group V CTLs.^[44]^ The lower lobe is constituted of an antiparallel β-sheet formed by N and C-terminal β-strands β1 and β5 and two flanking α-helices α1 and α2. This arrangement effectively brings the N- and C-terminus of the CTLD close together, ensuring a similar ‘loop-out’ orientation with respect to the plasma membrane for all CLRs independent of their topology.^[11]^ The upper lobe contains a three-stranded antiparallel β-sheet formed by β-strands β2, β3, and β4 and the long loop region, which can be partitioned by additional secondary structural elements into segments, which have been referred to as long and short loop for some CTLs.^[13,45]^ The upper β-sheet is part of the hydrophobic core containing a remarkable number of tryptophans, one of which is part of the conserved WIGL motif (Figure 1b). Overall, the CTLD can harbor up to four Ca^2+^ sites (I–IV) whose occupancy varies across CLRs. While site IV is positioned between α2, β1, and β5 and is rarely occupied, sites I–III are located in the long loop region, with site II functioning as canonical CBS.^[3]^ In general, the long loop region is conformationally flexible and highly variable in sequence (Figure 1c). Since it provides ligand specificity beyond monosaccharide recognition and impacts the stability and dynamics of the whole CTLD, the long loop region has been suggested to act as an independent part of the CTLD for adaptive evolution alongside diverging ligands and functions.^[46]^

### Canonical Glycan Binding

**Figure 2:**
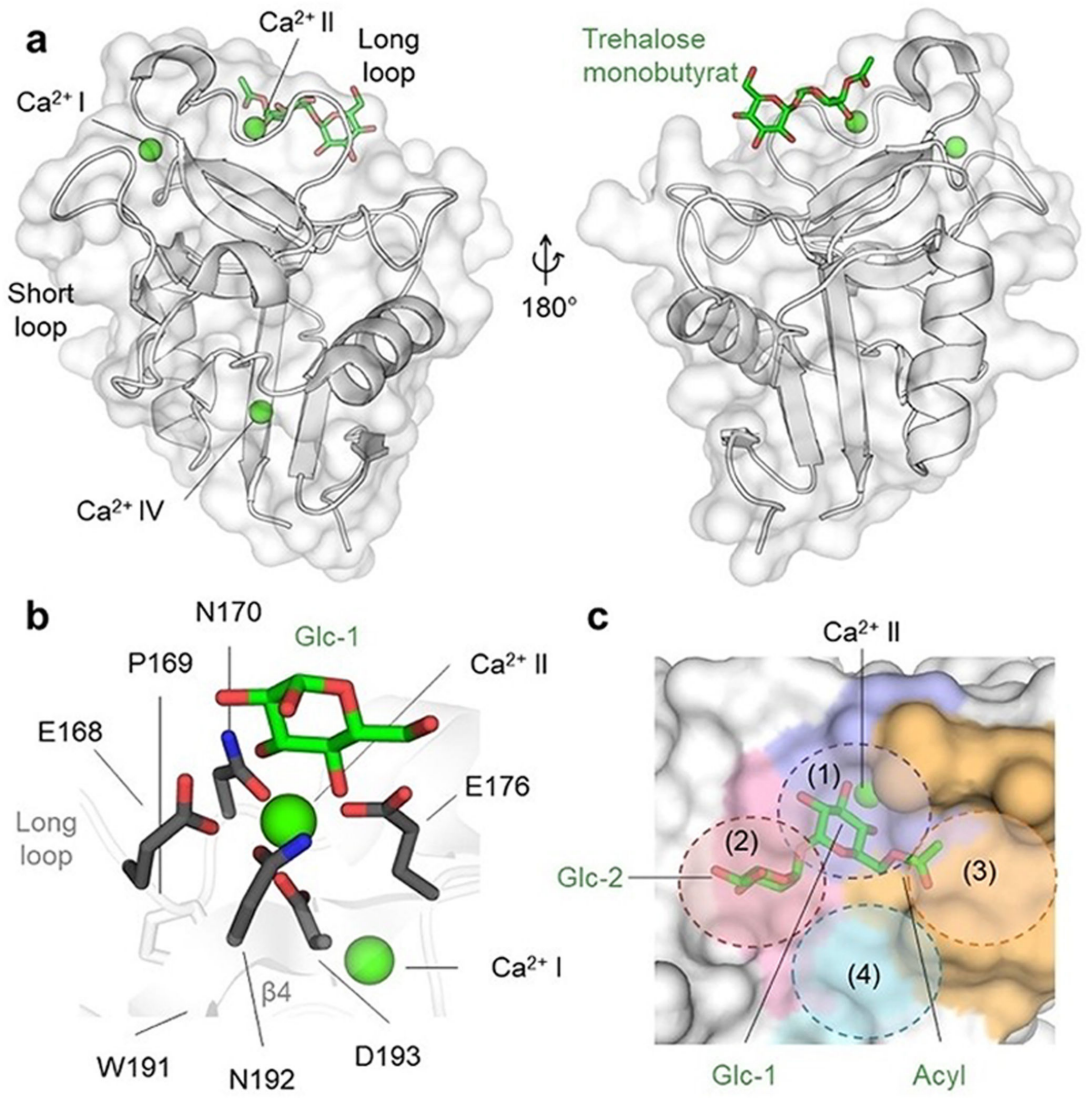
Canonical glycan binding at Ca^2+^ site II and the extended canonical CBS using glycolipid recognition by bovine mincle (PDB ID: 4ZRV) as an example. (a) Canonical glycan binding is dependent on Ca^2+^ site II in the long loop region. Bovine mincle in complex with trehalose monobutyrat is shown. Ca^2+^ ions are shown as green spheres. (b) The EPN motif (E168, P169, N170) together with the WND (W191, N192, D193) and E176 mediate interaction with the first glucose (Glc-1) moiety of trehalose monobutyrat via coordination of Ca^2+^ in Ca^2+^ site II together with 3- and 4-OH of Glc-1. (c) Extended glycolipid binding site of mincle. The primary carbohydrate site (1) coordinates Glc-1, as shown in (b), while the proximate carbohydrate site (2) interacts with Glc-2. The acyl chain of the trehalose monobutyrat is not fully resolved but points towards a hydrophobic grove designated as the primary lipid site (3). A second lipid site (4) was also proposed.^[54–57]^

In Ca^2+^-dependent CTLs, the long loop region harbors up to three Ca^2+^ sites (I–III) that coordinate Ca^2+^ ions *via* amino acids containing carbonyl sidechains. Although the occupancy of Ca^2+^ sites I–III varies between different CLRs, canonical carbohydrate binding is exclusively mediated *via* Ca^2+^ site II (Figure 2a).^[47]^ Together with a triad of residues, Ca^2+^ site II confers specificity towards different monosaccharide species within the CBS and couples Ca^2+^ binding to the hydrophobic core *via* the conserved WND motif (Figure 1b). Whereas the EPN motif mediates the Ca^2+^-dependent binding of mannose/fucose/glucose, the QPD motif allows the binding of galactose-related carbohydrates (Figure 2b). While E/D and N/Q residues coordinate Ca^2+^ together with vicinal hydroxy groups of the carbohydrate ligand, the central proline in *cis* configuration stabilizes the Ca^2+^-bound loop conformation.^[48,49]^ Further, selectivity towards certain monosaccharides is determined by additional structural elements of the canonical CBS, as illustrated by the differential recognition of fucose by DC-SIGN and the closely related DC-SIGNR or the increased affinity of ASGPR for N-acetylgalactosamine compared to galactose.^[26,50,51]^ Nevertheless, although Ca^2+^-dependent carbohydrate binding is generally considered specific, the flexibility of the long loop and solvent exposure of the CBS result in high entropic and desolvation costs, consequently leading to low millimolar affinities and high promiscuity for monosaccharide-CLR interactions.^[52,53]^

In a biological context, effective glycan recognition is accomplished by interactions with larger oligosaccharides and glycoconjugate structures.^[25]^ While a core monosaccharide anchors the ligand at Ca^2+^ site II, proximal amino acids establish contacts with additional carbohydrate residues, as well as conjugated peptides and lipids, increasing affinity of the interaction.^[58]^ This is particularly well illustrated by the dependency of mincle-induced signaling on oligosaccharides diesterized with branched fatty acids, such as trehalose dimycolate (TDM) and phenolic glycolipid-III (PGL-III).^[57,59,60]^ Several X-ray crystallographic and NMR studies of human and bovine mincle indicated that the interaction with these glycolipids involves Ca^2+^-dependent binding of the first glucose, an additional proximal binding site for the second glucose, as well as a hydrophobic grove for lipid binding^[54–57,61,62]^ (Figure 2c). Since these extended recognition interfaces are less conserved, they also provide selectivity, even between closely related CLRs, such as DC-SIGN and its mouse homologs SIGN-R1, 2, 3, 4, 5, 7, and 8.^[63–65]^ As documented by several examples, including the selectins, DC-SIGNR, MGL, mouse DCIR2, DCAR, and BDCA-2, this principle is common among Ca^2+^-dependent glycan binding CLRs and has been exploited for the design of glycan-drug-conjugates, also referred to as carbohydrate-based glycomimetics.^[6,30,62,66–71]^

### Conformational Plasticity

Early after the first crystallographic studies on Ca^2+^-binding CTLs, conformational plasticity has been suggested to play a major role in the modulation of Ca^2+^-dependent ligand binding and release.^[48,49]^ Apart from acting as a co-factor in carbohydrate binding, the Ca^2+^ sites in the long loop region mediate conformational change of the CTLD fold in response to reduced Ca^2+^ concentrations and acidic pH. In the absence of Ca^2+^ in Ca^2+^ site II, *cis-trans* bond of the EPN/QPD motif has been proposed to constitute a slow conformational switch that restricts ligands from rebinding.^[48,49]^ Allosteric cooperativity in CLRs with accessory Ca^2+^ sites I and III, such as MGL and ASGPR, results in higher-order Ca^2+^ -dependency and sharp transitions between active and inactive states.^[35,72,73]^ In mincle and DC-SIGNR, loss of an accessory Ca^2+^ under acidic conditions leads to conformational change in the CBS, restricting ligand binding under these conditions.^[54,74–76]^

Notably, the presence of the amino acids necessary for Ca^2+^ -coordination in Ca^2+^ sites I and III does not necessarily imply occupancy of these sites, nor does it suggest functional similarity to other CTLs. Residues not directly involved in Ca^2+^ -coordination, even peripheral ones, might affect the biochemical properties of Ca^2+^-dependent CTLs.^[17]^ For example, studies on the murine DC-SIGN homologs indicated differential response to varying pH.^[63,65]^ While all share the Ca^2+^-binding motives for Ca^2+^ sites I, II, and III, only DC-SIGN, SIGN-R1, 2, 3, and 7, showed reduced carbohydrate binding under acidic conditions. X-ray crystallographic structures of SIGN-R1 suggested Ca^2+^ sites I and III to be unoccupied due to a salt-bridge stabilized ‘closed’ long loop conformation that was previously observed in DC-SIGNR.^[64,76]^ SIGN-R5 binding was not affected by pH, and SIGN-R8 increased binding in response to acidification by an unknown mechanism.^[63]^ CTLs lacking the residues for accessory Ca^2+^ binding frequently have basic amino acids pointing into the Ca^2+^ sites and might have evolved other regulatory mechanisms for ligand release.^[71,77]^ For instance, in human langerin, a single histidine in the CTLD has been shown to act as a pH sensor and a central hub for an allosteric network that modulates Ca^2+^ affinity.^[17,78]^

Beyond the CTLD, different mechanisms involving the stalk and adjacent domains have been described to modulate CLR function in response to changes in pH. In DC-SIGN, tetramerization necessary for multivalent binding is abrogated at acidic pH, enabling ligand release upon endocytosis into endosomal compartments.^[79]^ Complex stepwise rearrangements of the CTLDs and additional domains in MR and other CLRs of group VI lead to a transition from an extended to a tightly packed conformation upon acidification, likely impacting ligand release and MR-dependent antigen presentation.^[19,20,80,81]^ Likewise, pH and ionic strength-induced changes in the tertiary structure of the Ca^2+^-independent F-actin binding CLR DNGR-1 lead to a conformational change in its stalk region promoting the formation of a reduction insensitive dimer critical for antigen presentation.^[82]^

Multidomain conformational changes upon ligand binding to the CTLD are less well understood, and only a few CLR-ligand interactions have been studied in this regard. Prominent examples include the interactions of the selectins with their endogenous ligands. Binding of the sialyl Lewis^x^ oligosaccharide to E-selectin induces conformational change in the long loop region, translating into a hinge-bending motion of the CTLD relative to the EGF and the CR domains from a low affinity bent conformation to a high affinity extended conformation, providing detailed insights into the force-enhanced mechanisms underlying cellular adhesion in the context of leukocyte trafficking.^[15,18,83–86]^ In LOX-1, hinge dynamics around the dimer axis, observed in molecular dynamics simulations and X-ray crystallographic structures, have been suggested to impact the binding of oxLDL.^[87–89]^ For DC-SIGN, previous reports indicated contraction of the tetrameric extracellular domain by 5 nm upon Man_9_GlcNAc_2_ binding, resulting from flexibility of the linkage between CRD and stalk domain.^[90,91]^ Moreover, DC-SIGN targeted antibodies induced distinct endocytic mechanisms depending on their epitope at the DC-SIGN extracellular domain, suggesting translation of extracellular ligand binding across the plasma membrane.^[92,93]^

### Secondary Sites

Conformational plasticity is often connected to the exposure of additional ligand binding sites as well as subject to their influence.^[28,94–96]^ Also referred to as secondary sites, these additional sites can display distinct structural and physicochemical features, enabling interactions with ligands other than the canonical primary site ligands. Since interactions at secondary sites can also have differential effects on protein function, they can provide an additional level of regulation and selectivity.^[27,97]^ Besides the canonical CBS, several CLRs recognize their ligands with non-canonical sites or possess additional secondary sites. Contrasting the extended recognition surface proximal to the CBS, these sites can be located remote from Ca^2+^ site II and are not dependent on a Ca^2+^-anchored carbohydrate as described for canonical glycan binding.

Some glycan-binding CLRs, such as dectin-1 and C-type lectin-like receptor 2 (CLEC-2], are devoid of any Ca^2+^-dependent canonical carbohydrate binding activity and bind glycans in non-canonical sites instead. While CLEC-2 interacts with a glycopeptide of podoplanin (PDPN) using a non-canonical site in the lower lobe of its CTLD, the CBS of dectin-1 has been proposed to be located in proximity of the canonical Ca^2+^ -dependent CBS of other CTLs.^[98–100]^ Other CLRs have potentially functional secondary sites in addition to their Ca^2+^-dependent canonical sites. For instance, X-ray crystallographic studies of SIGN-R1 have suggested a secondary site for dextran sulfates and other microbial glycans.^[64]^ Similarly, secondary sites for larger polysaccharides have been suggested for MGL, langerin, and DC-SIGN.^[16,101–103]^

**Figure 3:**
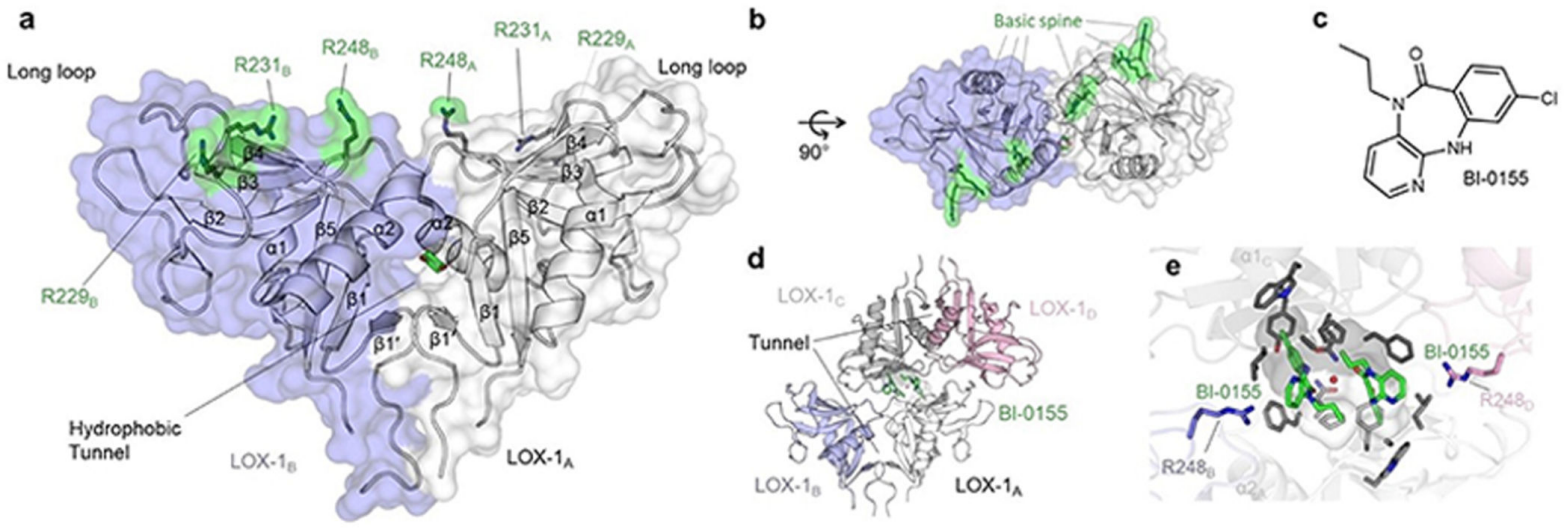
Structure and ligand binding sites of the LOX-1 CTLD. Front (a) and top (b) view of the X-ray crystallographic structure of the LOX-1 homodimer (PDB ID: 1YPQ). The hydrophobic tunnel bound to a dioxane molecule at the interface of the LOX-1 subunits was proposed to function as a lipid binding site. Arginine residues forming the basic spine necessary for oxLDL binding are highlighted as green sticks. Secondary structure numbering according to Zelensky and Gready.^[3]^ LOX-1 inhibitor BI-0155 (c) stabilizes the inactive tetramer state through head-to-head cross-linking of two LOX-1 dimers (PDB ID: 6TL9). (e) Close-up of the small molecule binding site. Two BI-1055 (green sticks) molecules bind two dimers LOX-1A/B and LOX-1C/D. The basic spine, as highlighted by residues R248B and R248D, is partly covered, preventing interaction with oxLDL.

Moreover, many CTLs have been reported to engage in well-defined Ca^2+^-independent protein-protein interactions with other CTLs, as well as with unrelated proteins, including immunoglobulins, major histocompatibility complex (MHC) class I-related proteins, or E-cadherin.^[104–106]^ This versatility for non-canonical ligand binding sites at the CTLD was recently highlighted in a meta-study across available X-ray crystallographic structures analyzing the frequency of contacts for those protein-protein interactions, indicating that nearly all parts of the fold are utilized to interact with proteins.^[107]^ Structural information on CTLs engaging in both protein-protein interactions and glycan binding is sparse but has been proposed, for example, for dectin-1 and DC-SIGN.^[108–110]^ An extreme case of a multiligand CLR is LOX-1.^[111–113]^ Two distinct putative binding sites have been described for the recognition of either lipids or proteins. First, a hydrophobic tunnel at the CTLD homodimer interface was suggested to interact with lipophilic ligands, including phospholipids and statins.^[40,114–116]^ Second, a basic spine formed by arginine residues diagonally crossing the dimer surface has been identified as the binding site for amphipathic α-helices on the surface of oxLDL particles (Figure 3a and b).^[41,87,88,117–119]^

Alongside their diverging ligand preferences, secondary sites have been frequently associated with increased selectivity and druggability compared to their canonical counterparts.^[97,120,121]^ Different approaches revealed secondary binding sites for drug-like small molecules in several CLRs – with important implications for novel modes of modulating their function.^[12,30]^ Despite overall low predicted druggability for CLRs, acceptable hit rates were obtained from fragment screening and orthogonal validation, identifying secondary sites amenable to traditional medicinal chemistry approaches.^[14,122–128]^ Likewise, high-throughput screenings (HTS) yielded biologically active and chemically tractable lead structures.^[89,129–131]^ Follow-up studies and mode of action analysis suggested some of these secondary sites to impact CLR function *via* unprecedented mechanisms, including allosteric modulation of the canonical CBS and changes in quaternary conformation.^[13,89,130–135]^ For instance, a recently identified small molecule ligand was shown to inhibit LOX-1-dependent oxLDL uptake by cross-linking two dimers in a head-to-head fashion, sterically blocking the basic spine region, and locking the receptor in an inactive tetramer state (Figure 3c, d and e).^[89]^ Consequently, apart from their biological role, secondary sites have become increasingly relevant in the context of CTL-targeted drug discovery.^[30]^

In the following examples, we highlight how the discovery of secondary sites by fragment-based drug discovery has opened new routes for allosteric modulation of DC-SIGN and langerin. Moreover, we summarize findings on an unusual secondary carbohydrate binding site in MGL, a CLR that recently sparked pharmaceutical interest.^[136]^ Finally, we discuss the molecular determinants of the non-canonical Ca^2+^-independent carbohydrate interactions of dectin-1 and CLEC-2 and touch on protein-protein interactions of CLRs by the example of NKG2D.

### Example 1: DC-SIGN

DC-SIGN is a Ca^2+^-dependent glycan binding CLR critical for innate to adaptive immunity.^[26,137,138]^ Expressed on antigen presenting cells (APCs), DC-SIGN facilitates cellular adhesion of migrating DCs and the formation of the immunological synapse, as well as pathogen recognition, subsequent internalization leading to antigen presentation and modulation of signaling pathways, contributing to immunity or tolerance.^[5,22,23,139–149]^ Apart from its protective role, DC-SIGN has attracted significant attention due to its interaction with HIV-1, proposedly leading to dissemination of the virus and *trans*-infection of CD4^+^ T cells.^[150–153]^ Similar involvement of DC-SIGN has been suggested in the disease progression of several pathogens, including Ebola virus, dengue virus, hepatitis C virus, measles virus, *Mycobacterium tuberculosis*, and *Helicobacter pylori*.^[154–160]^ Recent findings suggesting DC-SIGN to promote the severity of COVID-19 by acting as a co-receptor for SARS-CoV-2 have further spurred pharmaceutical interest in developing small molecules selectively targeting this CLR.^[161–167]^

**Figure 4:**
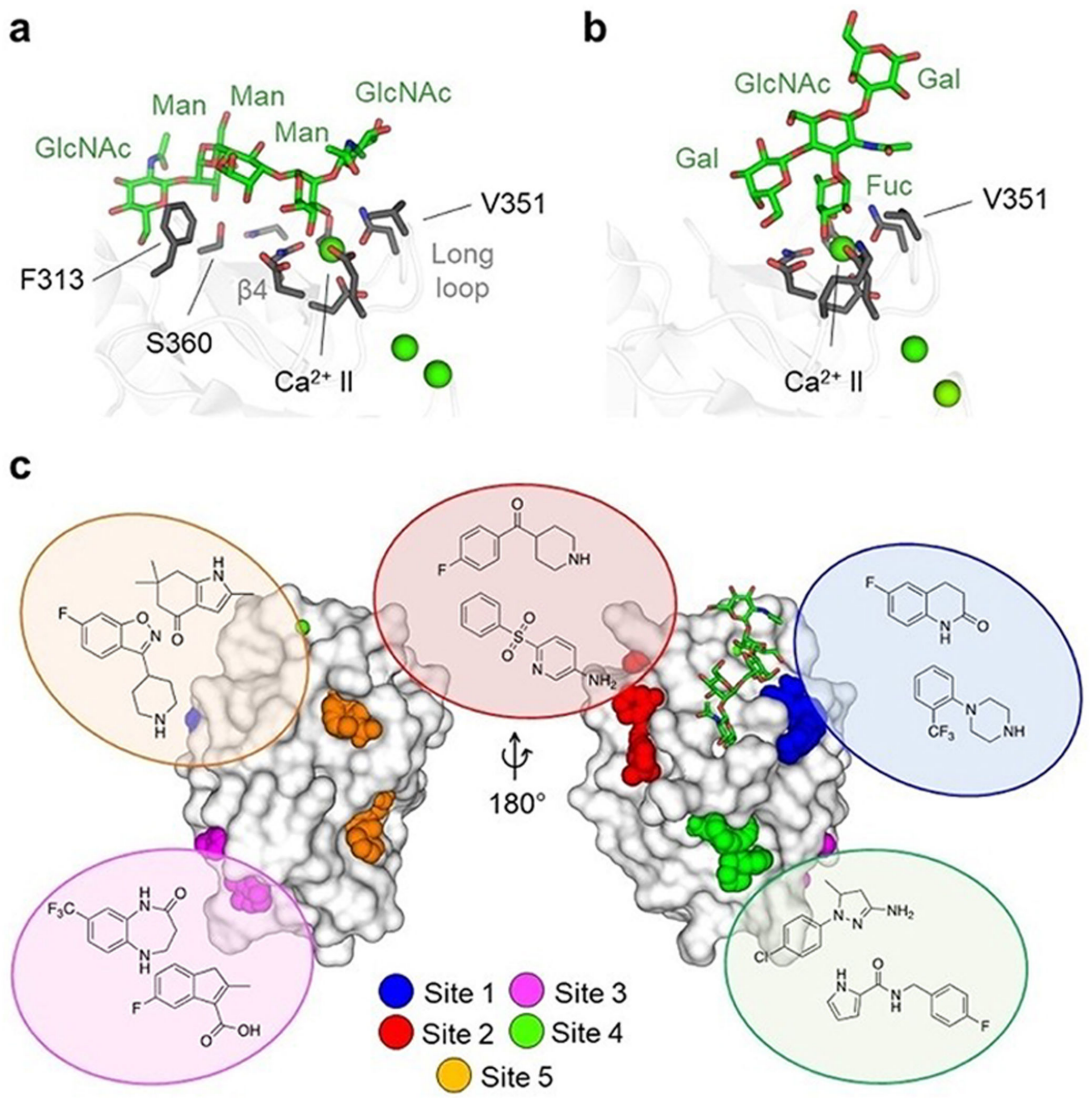
Canonical glycan binding and secondary sites in DC-SIGN. (a) High-mannose type oligosaccharide recognition at the extended canonical CBS using the X-ray crystallographic structure of DC-SIGN in complex with GlcNAc_2_Man_3_ (PDB ID: 1K9I). Residues involved in Ca^2+^-coordination of the central mannose (Man) and formation of the extended canonical CBS for interactions with the oligosaccharide are shown as sticks. Residues F313, S360, and V351, which are mostly responsible for forming additional interactions, are highlighted. Ca^2+^ ions are shown as green spheres. (b) Fucose-type oligosaccharide recognition at the extended canonical CBS using the X-ray crystallographic structure of DC-SIGN in complex with lacto-N-fucopentaose III (PDB ID: 1SL5). Residues involved in Ca^2+^-coordination and formation of the extended CBS for interactions with the oligosaccharide are shown as sticks. V351 is highlighted as a contributor to the recognition of the central fucose (Fuc). Ca^2+^ ions are shown as green spheres. (c) Proposed druggable secondary sites in DC-SIGN (PDB ID: 1K9I) and examples of fragment hits for each site. The GlcNAc_2_Man_3_ oligosaccharide indicates the canonical CBS. Figure adapted from Aretz et al.^[124]^

The CTLD of DC-SIGN contains three occupied Ca^2+^ sites (I–III) and an EPN motif, mediating canonical Ca^2+^-dependent interaction with high-mannose structures, often found on pathogens, as well as fucosylated oligosaccharides, such as Lewis-type antigens^[24,26,168,169]^ (Figure 4a and b). Since monosaccharide binding by DC-SIGN is of low affinity, oligosaccharide recognition depends on additional contacts with residues in the extended canonical CBS, including F313, V351, and S360.^[25,146,168]^ This recognition is significantly potentiated by avidity, arising from the multivalent presentation of the ligand, along with stalk-dependent tetramerization and clustering of the receptor in the plasma membrane.^[34,90,144,170–172]^ Due to the distinct spatial organization of the glycan ligands and differences in the topological arrangement of the CTLD between CLRs, multivalency additionally confers selectivity to the otherwise promiscuous nature of monovalent DC-SIGN-carbohydrate interaction.^[90,91,173–175]^

Building on these observations, many recent reports focused on the development of mannose-based glycomimetics exploring the vicinity of the canonical CBS, as well as multivalent presentation of DC-SIGN ligands on high-valency scaffolds, such as liposomes, dendrimers, poly-L-lysin conjugates and gold nanoparticles.^[30,52,162,165,167,173,176–178]^ Pioneering work utilizing structure-based design principles yielded monovalent glycomimetics based on an α1-2-linked pseudo-dimannoside scaffold and a triazole-substituted mannoside with low micromolar affinity for DC-SIGN and full selectivity over the potential off-target CLR langerin.^[62,162,179,180]^ However, multivalent display of these ligands was necessary to exert significant biological activity in cell-based assays, highlighting the pitfalls of targeting the shallow and hydrophilic canonical CBS of DC-SIGN.^[162,164,181]^

Considering the challenges of designing carbohydratebased drugs, HTS studies have explored non-carbohydrate drug-like compounds as alternative DC-SIGN inhibitors.^[129]^ Pyrazolone and quinoxalinone scaffold-based compounds with low micromolar affinities were identified that could later be optimized to yield compounds able to block multivalent interactions with nanomolar activity in cell-based assays.^[130]^ As these compounds lacked functional groups to complex Ca^2+^ ions, a CBS-independent allosteric mechanism was suggested.^[52]^

More recently, fragment screening studies identified several fragments with micromolar affinity and gave detailed insight into available secondary sites in DC-SIGN.^[122–125,128]^ In an explorative study, rigorous orthogonal screening and validation, including binding site mapping by ^1^H-^15^N heteronuclear single quantum coherence (HSQC) NMR in combination with computational tools, revealed five fragment binding sites (1-5) that could accommodate drug-like molecules^[124]^ (Figure 4c). While no hits were found for the canonical CBS, highlighting its low druggability, fragments bound in its vicinity in sites 1 and 2 around the long loop region. Since several of these fragments resembled substructures of the previously described quinoxalinone ligands, targeting these sites was suggested as a viable strategy to interfere with glycan binding by steric repulsion.^[124,133]^ Moreover, site I was later suggested to be targetable as a starting point for the design of glycomimetics.^[62,162]^ Sites 3–5 were found to be located remote from the canonical CBS in the lower lobe of the CTLD and accounted for more than half of the validated fragment hits. Although calculated druggability scores were rather low for these sites, single residues, in particular M270 in site 4, scored high in cryptic site predictions, suggesting respective sites to act as inducible sites that expand upon ligand binding.^[124,182]^ Moreover, the conformational plasticity of site 4 was later found to enable interaction with the p-hydroxymethylenebenzyl substituent of a glycomimetic ligand, effectively cross-linking two DC-SIGN CTLDs in the X-ray crystallographic structure.^[179]^

**Figure 5:**
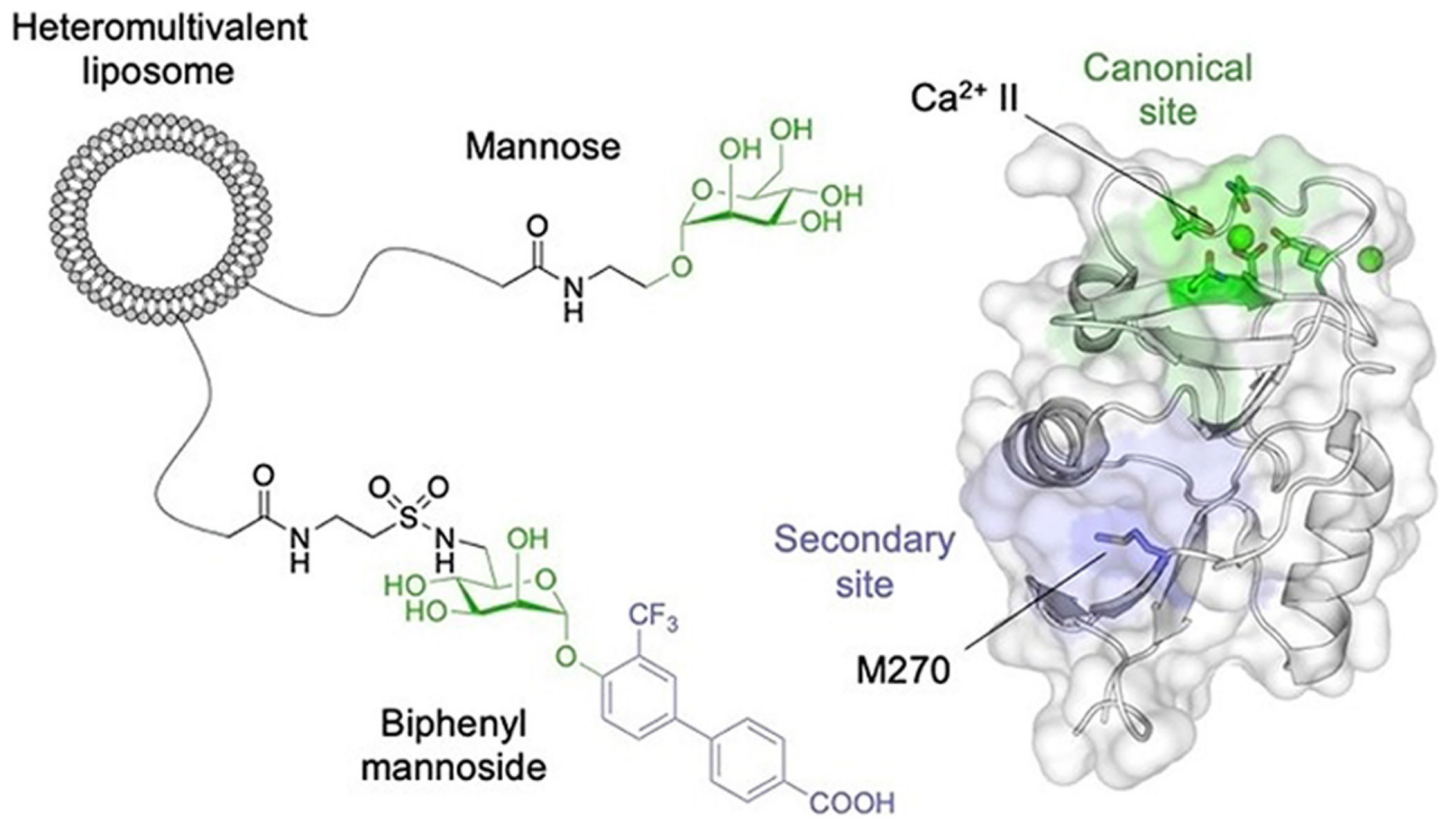
Proposed mechanism of selectivity of heteromultivalent liposomes carrying mannose and a biphenyl mannoside for DC-SIGN (PDB ID: 1SL4). While the biphenyl moiety of the biphenyl mannoside interacts with a previously identified secondary site centered by residue M270 (shown in blue), the mannose moiety predominantly binds to the canonical CBS of DC-SIGN (shown in green). This leads to chelation-derived avidity enhancement and allosteric activation of the canonical CBS or its associated Ca^2+^ sites, driving selectivity of the biphenyl mannoside for DC-SIGN.^[13]^

Notably, these studies did not only identify druggable secondary sites but also suggested allosteric modulation due to long-range perturbations observed in ^1^H-^15^N HSQC NMR experiments.^[124,183]^ This notion was further supported by a recent serendipitous finding, identifying a biphenyl-substituted mannoside glycomimetic that allosterically activated the canonical CBS of DC-SIGN.^[13]^ Originally designed to bind langerin, the ligand displayed striking selectivity for DC-SIGN expressing cells, when co-presented with mannose or fucose on heteromultivalent liposomes (Figure 5). Biophysical, mutational, and computational analysis revealed a dual binding mode, with the mannose scaffold predominantly binding to the canonical CBS of DC-SIGN and the biphenyl moiety interacting with a secondary site in a Ca^2+^-independent manner. Centered by residue M270, this site corresponds to the previously described fragment site 4 and was additionally computationally and experimentally proposed to exert long-range allosteric control over the canonical CBS of DC-SIGN.^[13,124]^ Together with chelation-derived avidity enhancement, allosteric activation of the canonical CBS or its associated Ca^2+^ sites was found to drive enhanced selectivity of the biphenyl mannoside for DC-SIGN.^[13]^

Although the pathways underlying this mechanism remain to be elucidated, these studies provided evidence for at least one secondary site being able to modulate DC-SIGN in an allosteric manner. The described ligands are still of low affinity but could serve as starting points for lead-like compounds, avoiding challenges frequently observed during the design of CBS-targeted glycomimetics. Finally, while the existence of non-canonical interactions of endogenous ligands with DC-SIGN has been speculated, the biological function of the described fragment sites and the allosteric network are unknown.^[30]^ This is particularly interesting in the context of the conformational plasticity and the ligand-dependent signaling activity observed for DC-SIGN.^[22,23,153]^

### Example 2: Langerin

**Figure 6:**
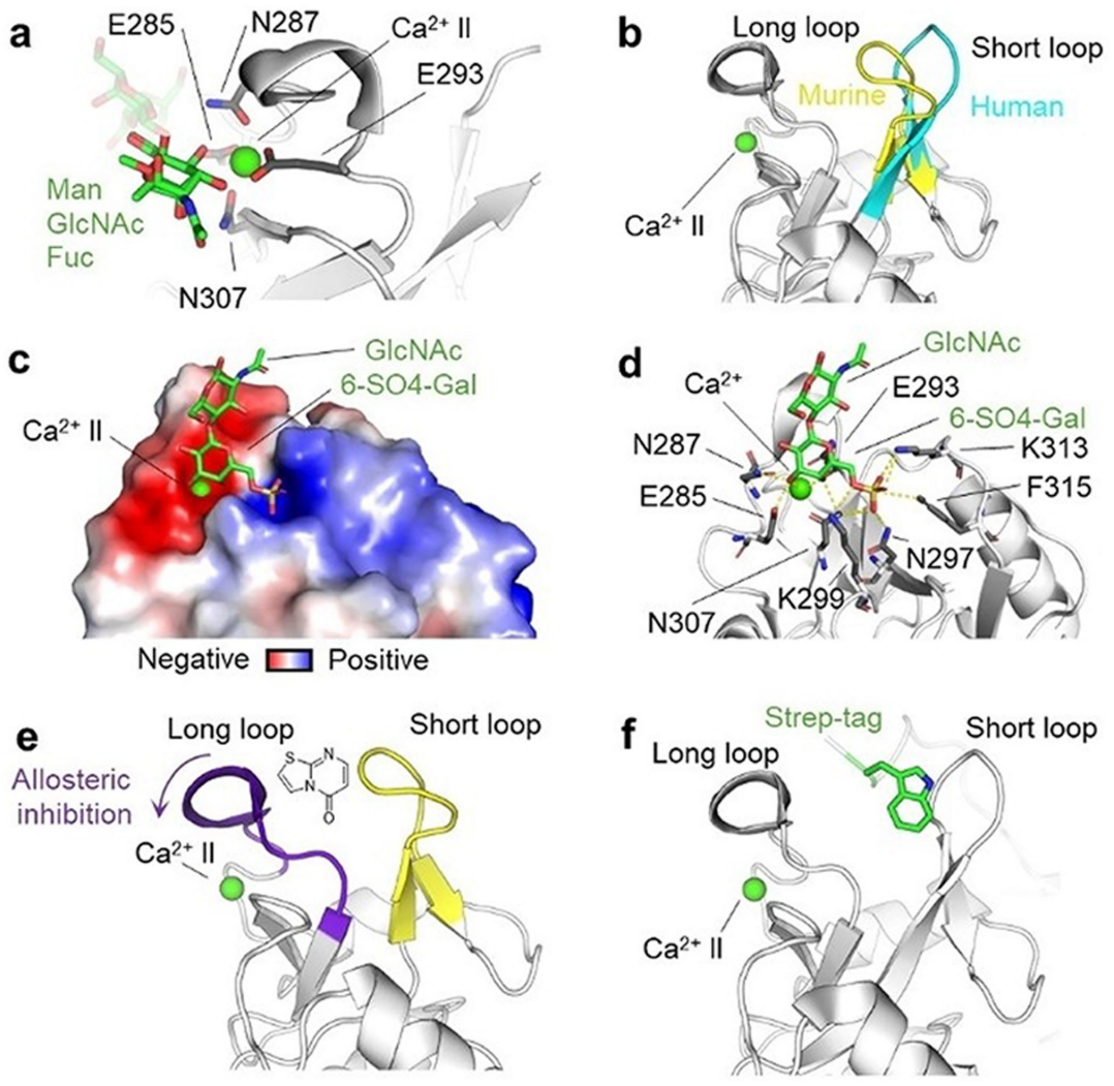
Carbohydrate binding mode, function of the loops, and secondary binding sites in langerin. (a) Overlay of mannose, glucosamine, and fucose (in Blood group B trisaccharide) interacting with langerin. The hydroxy groups coordinating the Ca^2+^ are all in equatorial configuration (PDB ID: 3P7G, 4N32, 3P5G). Ca^2+^ is shown as a green sphere, and the residues coordinating Ca^2+^, E285, N287, E293, and N307 are shown as sticks. (b) The short loop of langerin differs in conformation between human (blue) and murine (yellow) homologs (PDB ID: 3C22, 5 K8Y). (c) The surface charge on langerin shows the negatively charged canonical CBS and the positively charged extended canonical CBS, enabling salt bridges to form with 6-sulfated galactose. Surface charge was calculated using the APBS webserver.^[195]^ (d) Interaction details of 6-sulfated galactosamine; residues involved in the recognition are shown as sticks; salt bridges, and hydrogen bonds are shown in yellow (PDB ID: 3P5I). (e) Proposed allosteric inhibition ‘switch’ mechanism of thiazolopyrimidine binding to murine langerin. Adapted from Aretz et al.^[14]^ (f) The tryptophan (in green) of a Strep-tag from the neighboring langerin unit located at the cleft formed between the short loop and loop of langerin (PDB ID: 3P7H).

Langerin is a trimeric CLR expressed in Langerhans cells, where it plays a crucial role in the recognition and uptake of viral, fungal, and bacterial pathogens.^[184–187]^ The CTLD of langerin harbors a single Ca^2+^-binding site with an EPN motif for Ca^2+^ -coordination, implying specific recognition for mannose, glucose, and fucose through their equatorial hydroxy groups (Figure 6a). While murine and human langerin (Figure 6b) share a similar affinity for monosaccharides, the extended canonical CBS provides homolog-specific affinity and specificity for complex polysaccharides.^[188]^ Surprisingly, 6-sulfated galactose is recognized by langerin, highlighting the important role of the extended canonical CBS and how it affects not only oligosaccharide but also monosaccharide recognition.^[12,189]^ This enhanced affinity for sulfated glycans is primarily owed to the positively charged residues in the extended canonical CBS: K299 and K313 (Figure 6c and d).^[189]^ This charge-mediated preference for carbohydrate binding can be extended to substructures of glycosaminoglycans (GAGs) of limited length as heparin-like trisaccharides bind to the primary and extended sites.^[103]^ Interestingly, a longer heparin hexasaccharide was found to bind langerin *via* a distinct Ca^2+^-independent secondary site, as inferred from ^1^H-^15^N HSQC NMR.^[16,190,191]^ Computational modeling suggests that larger structures, such as heparin decasaccharide, interact with a remote secondary site located between two stalk regions of the trimeric langerin extracellular domain.^[191]^

Due to expression in Langerhans cells, langerin is a potential target for immunomodulating therapies. Carbohydrate-based sulfated glycomimetics were designed to expand into the extended canonical CBS.^[13,192,193]^ Building on the binding mode of monosaccharide (Figure 6a), a library of metal-binding pharmacophores was screened against langerin to replace the carbohydrate scaffold while keeping Ca^2+^-coordination, which led to the identification of drug-like fragments.^[194]^

Recently, fragment screening against murine langerin identified a series of thiazolopyrimidines as non-carbohydrate-based inhibitors.^[14,30]^ As demonstrated by an array of biophysical methods, these ligands were found to act as allosteric inhibitors, and their binding site was subsequently mapped to a cleft formed between the short and long loop (Figure 6e).^[132]^ Interestingly, the same cleft is occupied by a tryptophancontaining purification tag in the X-ray crystallographic structure of langerin (Figure 6f).^[45]^ In further support of the thiazolopyrimidine binding site being located in the cleft, single point mutations on the loop converted the human to the murine langerin with respect to their small molecule specificity (Figure 6b).^[132]^ Finally, trimeric langerin is able to engage in protein-protein interactions *via* its CTLD. Cryo-electron tomography demonstrated how the formation of Birbeck granules, an organelle specific to Langerhans cells, takes place by the arrangement of langerin trimers in a ‘honeycomb lattice’ mediated by an interface located in the short loop.^[196]^

Taken together, langerin harbors a hydrophilic canonical CBS and positively charged extended site as well as an allosteric secondary binding site able to accommodate non-carbohydrate modulators. An additional remote secondary binding site was suggested to harbor large GAGs. These discoveries could provide new strategies for the development of small-molecule ligands.

### Example 3: MGL

MGL is a trimeric Ca^2+^-dependent CLR predominantly expressed in macrophages and DCs. Its involvement in tumor immune invasion, progression, and modulation of the immune response within the tumor microenvironment is well-documented.^[197–199]^ Murine MGL has two isoforms, mMGL1 and mMGL2. Their expression profiles differ in tissues, suggesting separate functions.^[200]^ Whereas mMGL1 has distinct carbohydrate specificity, recognizing the Lewis-type oligosaccharides Lewis^X^ and Lewis^A^, mMGL2 is similar to hMGL with respect to its preferred carbohydrate epitope and therefore considered the functional hMGL homolog.^[201]^ MGL recognizes glycans containing terminal galactose or galactosamine, with higher affinity for the latter.^[202]^ Notably, MGL is involved in the detection of various pathogens, including *Staphylococcus aureus, Campylobacter jejuni, Klebsiella pneumoniae, Neisseria gonorrhoeae, Bordetella pertussis*, and *Mycobacterium tuberculosis*.^[203–206]^

MGL possesses a QPD motif for canonical Ca^2+^-dependent recognition of galactose-related glycans. While MGL typically features two Ca^2+^ sites, X-ray crystallographic structures (*e. g*., PDB ID: 6PY1 and 6 W12) revealed additional Ca^2+^ ions, postulated to enhance protein stability.^[207]^

**Figure 7:**
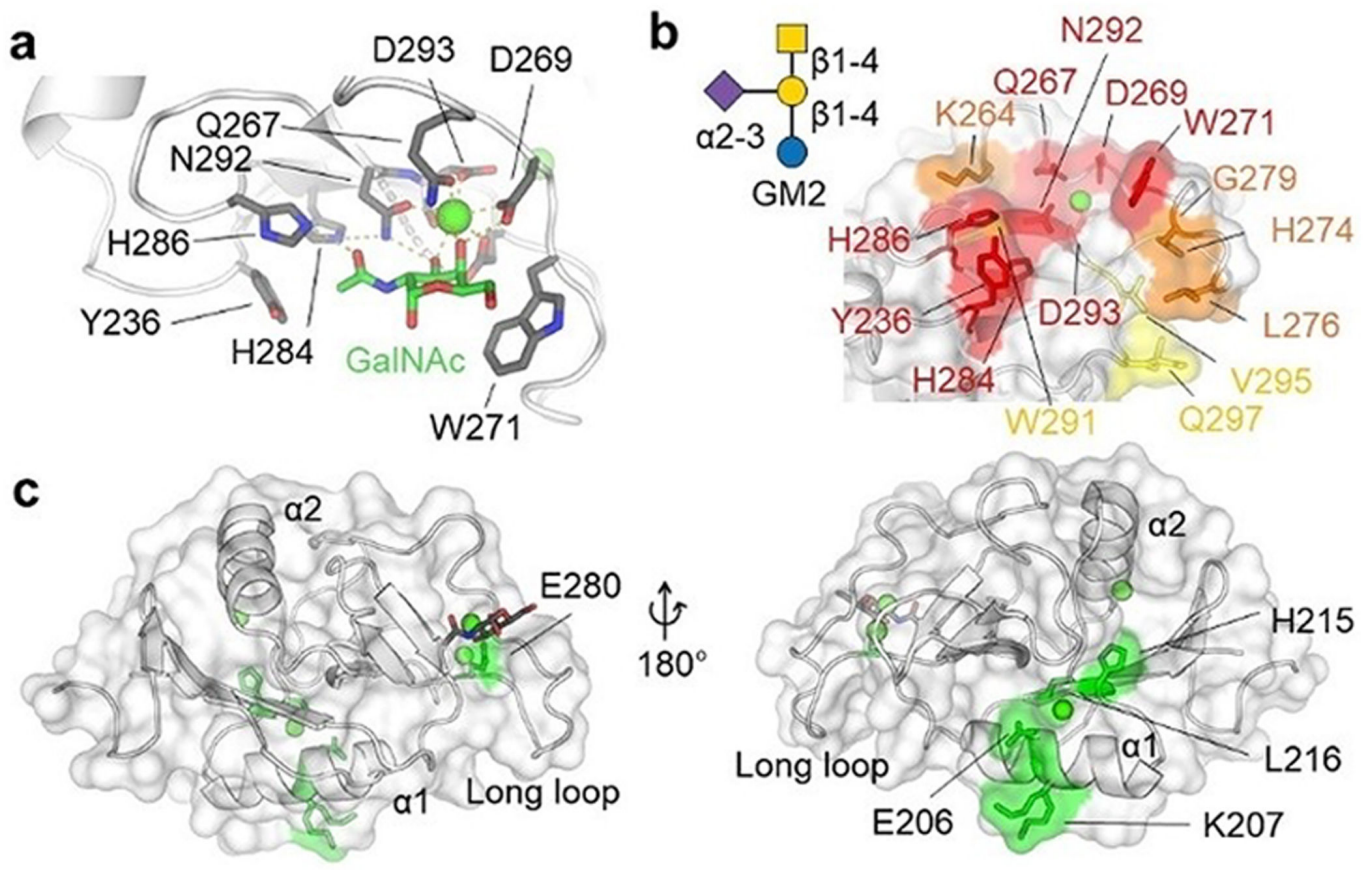
Carbohydrate binding mode, extended canonical CBS, and remote secondary site of the MGL CRD. (a) X-ray crystallographic structure of MGL bound to N-acetylgalactosamine (PDB ID: 6PY1). Residues related to carbohydrate recognition and Ca^2+^-coordination are shown as sticks. Ca^2+^ is shown as a green sphere. (b) Chemical shift perturbations (CSPs) in ^1^H-^15^N HSQC spectrum induced by GM2 are mapped on PDB ID: 6PY1. Adapted from Diniz et al.^[68]^ The canonical CBS residues are colored in red; the extended canonical CBS residues are colored in orange (CSP > 0.05 ppm) and yellow (CSP< 0.05 ppm). (c) Remote secondary site revealed by ^1^H-^15^N BEST-TROSY spectra of MGL. CSPs induced by *E. coli* R1 lipooligosaccharide are mapped on the CTLD of MGL (PDB ID: 6PY1). The CBS is shown on the left, and the remote secondary site is shown on the right. Residues inducing large perturbations (CSP>0.018 ppm) are shown in green.^[101]^

Several X-ray crystallographic structures of the MGL CRD are available, revealing the interaction details between its canonical CBS and its carbohydrate ligands galactose and N-acetylgalactosamine. Preferential binding of the latter is primarily attributed to the hydrogen bond between the acetamide group and H286 (Figure 7a), which is not observed in the co-crystal structure of galactose with MGL.^[207]^

Like langerin and DC-SIGN, MGL gains specificity and affinity for oligosaccharides from an extended recognition surface proximal to the canonical CBS. For mMGL1, a series of NMR experiments suggested the Lewis^x^ trisaccharide bind to MGL with its galactose moiety interacting with Ca^2+^ site II and fucose, establishing additional contacts with residues of the extended binding site.^[208]^ Similarly, for hMGL, a combination of saturation transfer difference NMR and ^1^H-^15^N HSQC NMR revealed that the N-acetylgalactosamine moiety of GM2 interacts with the canonical CBS, positioning the oligosaccharide to interact with the extended binding site (Figure 7b).^[68]^

Besides the extended canonical CBS, a remote secondary site was identified for MGL. N-acetylgalactosamine at high concentration could not significantly compete with the binding of *E. coli* R1 lipooligosaccharide, suggesting the recognition being independent of the canonical CBS.^[101]^ Notably, the D269H mutant in the QPD motif abolished Ca^2+^-coordination but retained the ability to bind this lipooligosaccharide. Mapping of CSPs observed in ^1^H-^15^N BEST-TROSY spectra upon addition of *E. coli* R1 to MGL revealed the binding site to be located in the lower lobe of the CRD of MGL, opposite to the canonical CBS (Figure 7c). This remote secondary binding site is attributed to the relatively open 3D organization of MGL-CRDs compared with other multimeric CTLs, such as langerin and mannosebinding protein, which adopt a compact arrangement of their CRDs with smaller distances between adjacent glycan binding sites.^[101,209,210]^

These binding sites offer a promising opportunity to develop ligands with enhanced selectivity towards MGL over other galactose-binding CTLs such as ASGPR.^[50]^ While the discovery of glycomimetics and other small-molecule ligands targeting MGL is still in its infancy, developing ligands specific for MGL, selectively binding the canonical CBS, the extended canonical CBS, or the remote secondary binding site, will help in understanding the biology of this CLR.^[136]^

### Example 4: Dectin-1

Dectin-1 is a glycoprotein mostly found on the surface of myeloid cells, where it serves as a non-opsonic receptor in fungal defense. The main ligands of dectin-1 are β-glucans, a pathogen-associated molecular pattern (PAMP) found in plants, bacteria, and primarily fungi.^[211]^ Beyond its important role as a β-glucan receptor, dectin-1 is increasingly recognized as an inflammatory regulator and for its involvement in adaptive immunity.^[212]^

**Figure 8:**
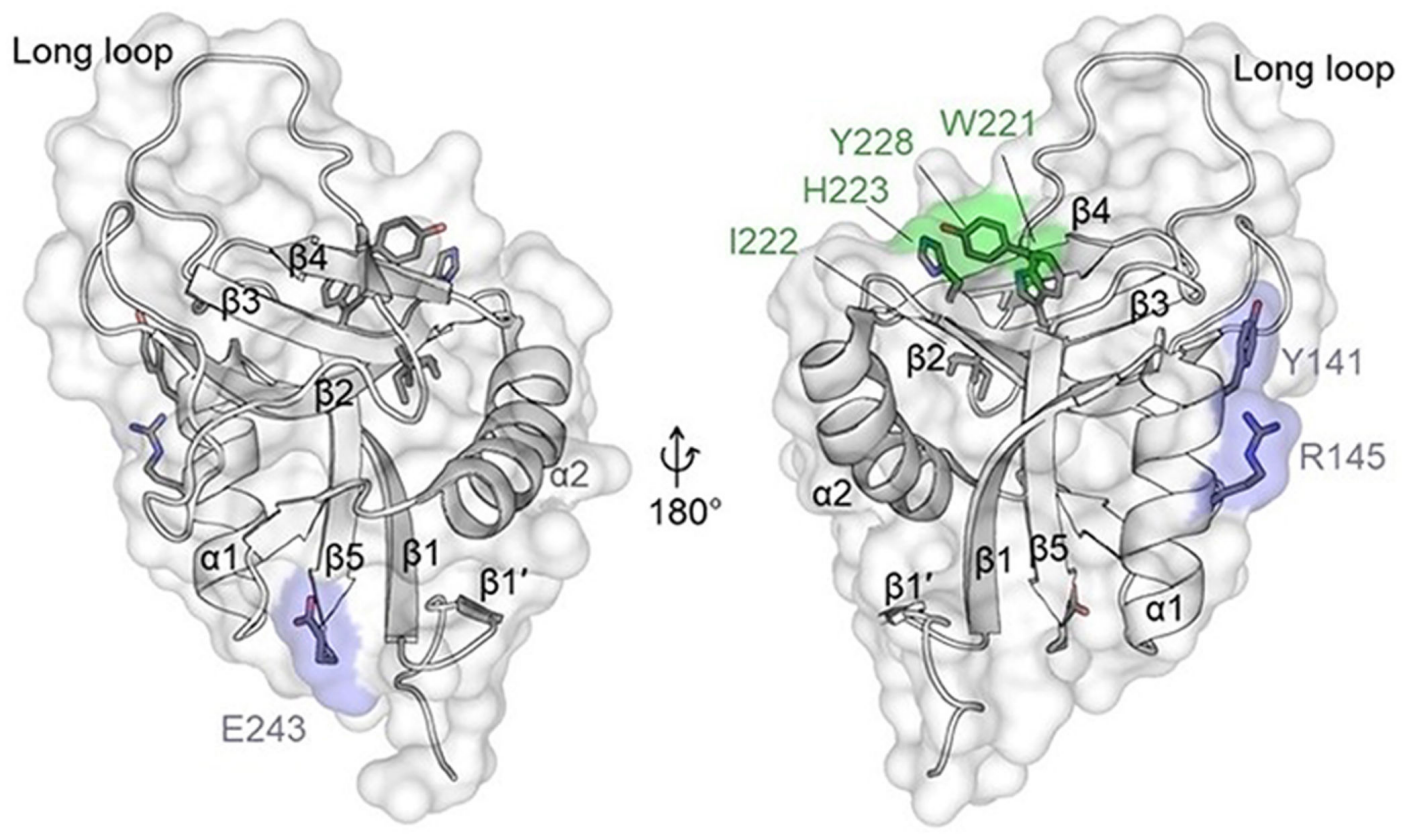
β-glucan binding site and proposed oligomerization site mapped on the murine dectin-1 CTLD (PDB ID: 2BPE). Amino acids of the hydrophobic group forming the putative non-canonical carbohydrate recognition site for β-glucans in the upper lobe of the CTLD are labeled green.^[99,100]^ Amino acids important for ligand-induced cooperative oligomerization of the CTLD are labeled in blue.^[100]^ Secondary structure elements were numbered according to the nomenclature proposed by Zelenksy and Gready.^[3]^

Structurally, dectin-1 is composed of a short intracellular domain that contains a hemITAM for downstream signaling linked to the extracellular CTLD *via* a helical transmembrane domain and a short stalk region. Of the four Ca^2+^-binding sites commonly found in CTLDs, dectin-1 only shows divalent metal coordination in site IV, which is located in the lower lobe.^[213]^ Like all group V CLRs, the protein lacks the canonical Ca^2+^ -binding motives in the long loop region along with the corresponding ability of canonical carbohydrate recognition (Figure 8).^[213]^ However, dectin-1 still serves as a pattern recognition receptor for glycans found in the cell wall of pathogens, specifically oligomeric β(1–3) and/or β(1–6) linked D-glucose, commonly referred to as β-glucans.^[211]^ The putative interaction site has been mapped by extensive mutagenesis to a binding interface located in the upper lobe of the CTLD. Three aromatic amino acids, W221, H223, and Y228, are essential for β-glucan binding and frame a shallow hydrophobic groove on the dectin-1 surface (Figure 8).^[99,100]^

Interestingly, dectin-1 is not the only CTLD-containing protein utilizing this binding site. The surface overlaps with the putative interface for non-canonical recognition of heparin and α2,3-linked N-acetylneuraminic acid on multi-antennary N-glycans by NKG2D/CD94, the extended canonical CBS for sialyl Lewis^x^ on E-selectin and the extended canonical CBS for GlcNAc_2_Man_3_ on DC-SIGN (Figure 4a).^[6, 168,214,215]^ While an X-ray crystallographic structure of laminaritriose in complex with murine dectin-1 CTLD exists, the observed pose of the ligand is considered to be physiologically not relevant.^[213]^ Indeed, it was found that β-glucans binding to dectin-1 have longer glucose backbones of at least 10 to 11 subunits or seven subunits when a β(1-6) linked glucose is introduced at the non-reducing end.^[216,217]^ The positive correlation of oligomer length and affinity was suggested to be due to increased secondary structure formation of the oligosaccharide.^[218]^ Binding studies with seaweed β-glucan laminarin have revealed that dectin-1 forms a protein-glycan complex with defined 4 : 4 stoichiometry by a cooperative oligomerization mechanism.^[100,213]^ Since oligomer formation can be reduced by mutagenesis of amino acids Y141, R145, and E243, located opposite to the ligand binding grove, oligomerization *via* direct protein-protein interaction has been proposed (Figure 8).^[100]^ Different fractions of laminarin can serve as either dectin-1 agonists or antagonists, independent of their affinity to the receptor. Anaya *et al*. were able to demonstrate that dectin-1 signaling is dependent on the formation of small receptor clusters, and the agonistic potential of the ligand appears to be determined by its size and higher-order structure.^[219]^

It is important to note that most studies using recombinant dectin-1 are conducted with murine dectin-1 (m-dectin-1) as recombinant production of human dectin1 (h-dectin-1) in *E. coli* is challenging.^[220]^ Even though the murine protein shows ~60 % sequence identity with the human homolog, the two homologs display different sensitivity to β-glucans of different valency.^[221]^ Interestingly, this difference is not mediated by the dectin-1 CTLD but depends on the intracellular domain of the protein, as shown by reciprocal mutagenesis.^[222]^ The murine CTLD is considered a reasonable model for β-glucan binding to dectin-1.^[213]^

In addition to β-glucans, multiple protein ligands for dectin-1 have been described, and dectin-1 has gained interest as a target in cancer therapy due to high expression of dectin-1 in tumor-associated macrophages that create an immuno-sup-pressive environment when activated by galectin-9.^[110,223–229]^ Although the exact binding mode of most protein ligands for dectin-1 is still an open question, the interaction of human dectin-1 with CLEC-2 has been mapped to a distinct interactions motif in the stalk of dectin-1 and binding sites of annexins and angiotensin II appears to be different from the binding site of β-glucan.^[110,224,226]^

Dectin-1 illustrates how a binding site distinct from canonical Ca^2+^-dependent carbohydrate recognition site mediates glycan binding and becomes the primary binding site for the role of dectin-1 as a pattern recognition receptor. Moreover, the existence of multiple protein ligands with potentially more distinct binding sites underlines the functional variability of the CTLD fold.

### Example 5: CLEC-2

The glycoprotein CLEC-2 is mainly expressed on platelets and associated with a set of diverse functions commonly performed by these cells.^[230]^ In addition to mediating platelet activation and aggregation in response to damage to the blood vessel wall, CLEC-2 is crucial for embryonic development, particularly for proper blood-lymph separation, and has been linked to pathophysiological processes like metastasis cancer and thromboinflammation.^[231–234]^

Besides an additional tightly packed 3_10_-like helix in the long loop region, the CLEC-2 CTLD adheres to the basic structure of other group V CLRs devoid of any Ca^2+^ binding sites. The CTLD is linked *via* a helical transmembrane domain region to the intracellular domain CLEC-2, which contains a hemITAM motif for signaling.^[235]^ Engagement of CLEC-2 leads to the formation of receptor clusters, which are cross-linked intracellularly by tyrosine kinase Syk. Upon phosphorylation of CLEC-2, Syk is activated *via* trans-autophosphorylation and triggers downstream signaling, leading to calcium signaling and platelet activation.^[236]^

**Figure 9:**
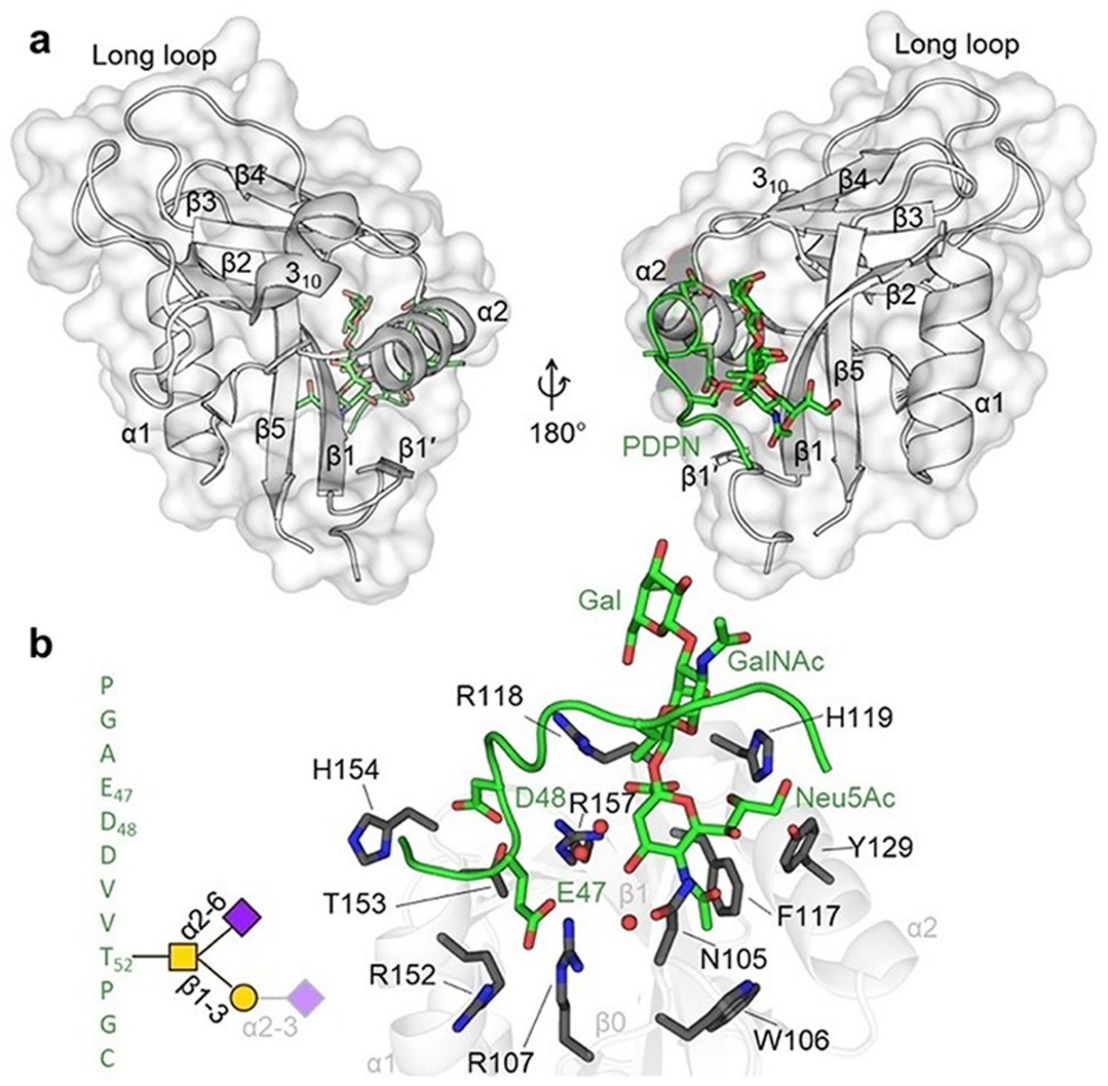
PDPN recognition of CLEC-2 via a non-canonical binding site. (a) Crystal structure of the CLEC-2 CTLD (white) in complex with PDPN-derived glycopeptide (green) (PDB ID: 3WSR) showing the binding site of PDPN located in the lower lobe of the CLEC-2 CTLD between α1, α2, β0, and β1. Secondary structure elements were numbered according to the nomenclature proposed by Zelensky and Gready.^[3]^ (b) Amino acids sequence of PDPN glycopeptide residues that show electron density in the crystal structure with di-sialylated core I O-glycan attached to T52 (the α2-3 linked N-acetylneuraminic acid was not resolved in the structure) (left) and close up of the PDPN binding site of CLEC-2 (right). The glycopeptide is mostly recognized via two acidic amino acids (E47 and D48) and the α2-6 linked N-acetylneuraminic acid. Amino acids of CLEC-2 that are important for the interaction are shown as sticks and labeled.

**Figure 10:**
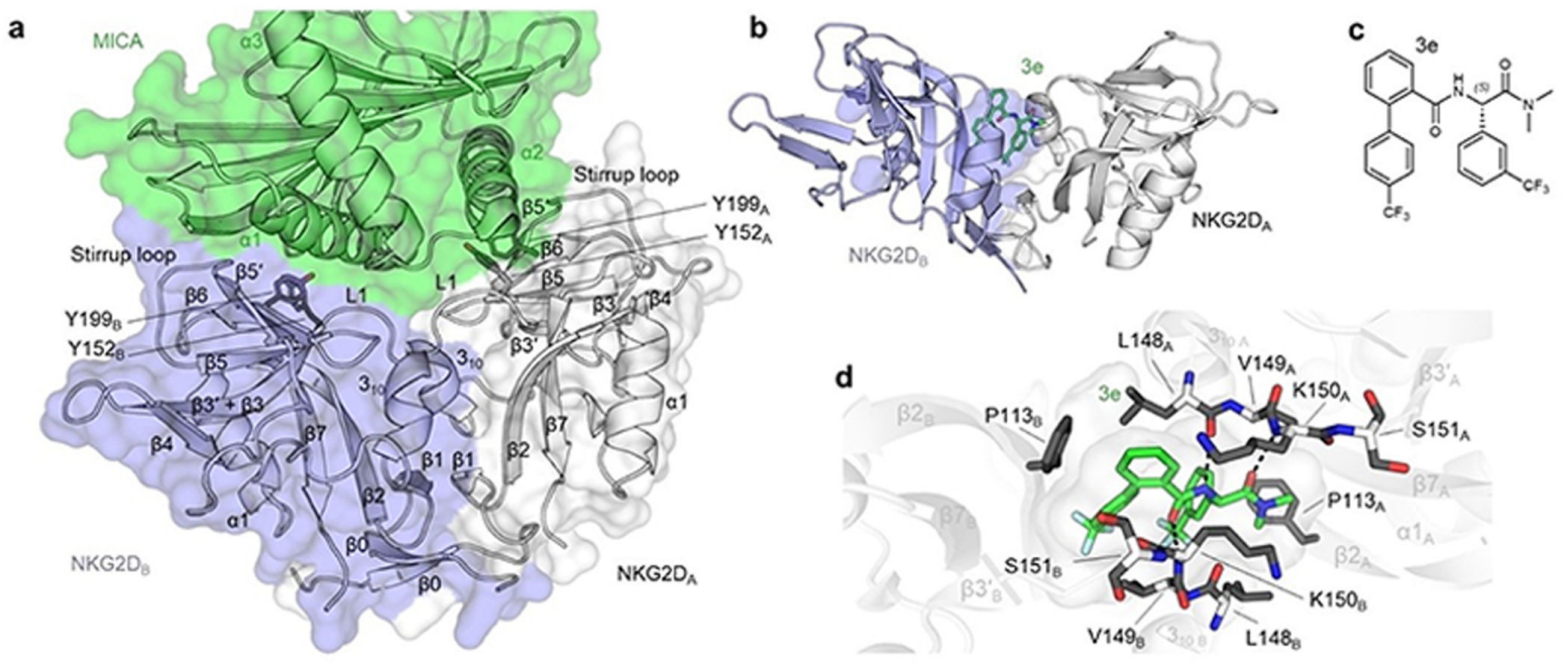
Distinct protein and small-molecular binding sites of NKG2D. (a) The NKG2D homodimer is blue and white in complex with green MICA (PDB ID: 1HYR). Secondary structure elements were numbered according to Li et al.^[105]^ Interaction hotspots Y152 and Y199 for the recognition of protein ligands by NKG2D are shown as sticks. (b) The NKG2D homodimer in complex with small-molecule ligand 3e (PDB ID: 8EA6) occupying a cryptic pocket in the interaction interface of the two subunits. (c) Chemical structure of NKG2D ligand 3e. (d) Close-up of the 3e binding site. The ligand forms hydrogen bonds with L148_A_, K150_A_, and K150_B_, replacing reciprocal backbone interactions of the unbound dimer. F113_B_ is the only sidechain affected by ligand binding and forms π–π stacking interactions with the phenyl rings of 3e. Hydrogen bonds are shown as dashed lines.

The first and, for a long time, only known endogenous ligand of CLEC-2 was the transmembrane receptor PDPN.^[237]^ The crystal structure of PDPN-derived glycopeptide in complex with CLEC-2 CTLD revealed its distinct binding site between helices α1 and α2, and β-strands β1, and β1’ (Figure 9a), a protein surface often utilized by group V CLRs proteins for dimerization as seen for NKG2D (Figure 10a & b).^[98]^ Two adjacent binding pockets of CLEC-2 enable the recognition of the PDPN peptide and its di-sialylated core I O-glycosylation. In the first interaction locus, two acidic side chains of PDPN are involved in electrostatic contacts with a large positively charged patch on the CLEC-2 surface formed by four arginine residues (R107, R118, R152, and R157]. Further, the α2-6-linked N-acetylneuraminic acid binds a proximal site *via* its carboxylic acid to R118, forms hydrogen bonds with H119 and Y129, and hydrophobic interactions with W106 and F117 (Figure 9b).^[98]^ Additionally, dectin-1 has recently been identified as an endogenous protein-ligand of CLEC-2. Dectin-1 is recognized by CLEC-2 *via* a similar binding motif as PDPN consisting of two acidic amino acids in the proximity of the same di-sialyl core 1 O-glycosylation located in the stalk region of dectin-1. Interestingly, dectin-1 causes platelet activation without triggering thrombus formation.^[224]^ Finally, the snake venom protein rhodocytin is an endogenous protein-ligand for CLEC-2 that triggers rapid platelet activation and aggregation.^[238]^ The interaction of the rhodocytin α-subunit and CLEC-2 is carbohydrate-independent as rhodocytin is not glycosylated but essentially utilizes the same surface as PDPN. The N-terminal loop of rhodocytin contains two acidic residues interacting with the same basic residues of CLEC-2 as PDPN. Second, a binding spot different from the N-acetylneuraminic acid interacting site, the C-terminal Y136 of rhodocytin interacts with CLEC-2 R118.^[98]^ CLEC-2’s direct implication in thromboinflammation, platelet exclusive expression pattern, and minimal involvement in hemostasis have led to CLEC-2 gaining traction as a promising target for novel antithrombotic drugs.^[230,234,239–241]^ Multiple attempts have been made to develop small molecule ligands for CLEC-2 to inhibit PDPN-CLEC-2 interaction. Three of the four published synthetic CLEC-2 ligands have been docked into the PDPN binding site, indicating a competitive inhibition of the interaction.^[242–244]^ Only cobalt hematoporphyrin was docked to a previously unidentified binding site consisting of N210, K211, and N120, potentially clashing with confirmed glycosylation of N120.^[245,246]^ Computational analysis of the CLEC-2 CTLD indicated the existence of four binding sites, of which one is predicted to be druggable.^[122]^ To sum up, CLEC-2 has three experimentally verified non-canonical interaction loci located in the lower lobe of the CTLD that allow for the adaptable recognition of endogenous glycoproteins and exploitation by an exogenous ligand. The physiological function of CLEC-2 depends on its ability to bind carbohydrates with a non-canonical interface remote from the canonical CBS.

### Example 6: NKG2D

NKG2D is a CTLD-containing protein mainly expressed on the surface of cytotoxic lymphocytes. It plays an important role in innate and adaptive immunity, coordinating the defense against infected or malignant cells.^[247,248]^ The known endogenous ligands of NKG2D display low expression on the surface of normal cells. Increased expression on distressed cells activates NKG2D signaling and leads to cytolysis.^[247]^ Downstream signaling, as well as expression of the distinct NKG2D agonists, has been discussed in recent reviews.^[248,249]^

The NKG2D homodimer is formed by two monomers *via* a complementary interaction interface in the CTLD. The structure of both monomers is very similar and, for the most part, follows the expected basic architecture of CTLDs. The NKG2D CTLD has the two conserved β-sheets and forms an extra β-strand (β0) at its N-terminus as well as an extra β-strand (β5’) in the long loop region that adds to the second canonical β-sheet and leads to the formation of ‘stirrup’ loop. Instead of the canonical α2-helix, it has a short 3_10_ helix (Figure 10a). Each monomer is stabilized by four intramolecular disulfide bridges and does not contain any of the canonical Ca^2+^ binding sites.^[105,250]^ A short stalk region and a transmembrane domain connect the CTLD to a short intracellular domain devoid of any signaling motif. Instead, the human NKG2D homodimer binds two DAP10 homodimers as signaling adaptors, forming a functional hexamer.^[251]^

Eight protein ligands for NKG2D have been identified so far, all sharing an MHC-I-like fold, possessing α1α2-platform domain. Crystal structures of several protein-ligand complexes give critical insights into the protein binding site of NKG2D. Each monomer of the NKG2D dimer contributes to the interaction with the L1 loop, the stir-up loop, and the β6-strand, creating a symmetric binding site. The complex is formed with a 2:1 stoichiometry, in which each of the NKG2D monomers binds to either the α1 or α2 domain of the MHC-I like ligand. The degeneracy of the NKG2D binding site allowing it to interact with multiple different protein surfaces, is mainly mediated by conserved hotspot interactions like Y152 and Y199 (Figure 10a). The distinct protein-protein interactions of NKG2D are not based on induced fit but on ‘rigid body adaptation’.^[250,252,253]^ In a meta-analysis of the crystal structures of CTLD-containing proteins involved in protein-protein interaction, Dohnálek and Skálová found that most protein-protein interactions are mediated by a surface in the upper lobe of the domain that overlaps with the protein-protein interaction interface of NKG2D.^[107]^ While the residues constituting this surface are not particularly well conserved between different CTLDs, amino acids mapping to the Y152 were found to be involved in protein-protein interactions in 15 CTLD crystal structures. Their analysis concluded that ~77 % of the CTLD surface is, to some extent, involved in the binding of protein ligands, highlighting the high adaptability of the CTLD fold, allowing for the recognition of a diverse array of ligands.^[107]^

Despite the lack of Ca^2+^ binding sites, NKG2D still was found to interact with carbohydrates. Heparin and α2,3-linked N-acetylneuraminic acid on multi-antennary N-glycans on transferrin bind to the NKG2D CTLD with low micromolar affinities.^[254]^ In both cases, Y152 and Y199 have been identified as essential for the interaction. In addition to being critically involved in protein-protein interaction interactions of NKG2D, Y152 maps the putative β-glucan binding site of dectin-1 and extended canonical CBS of E-selectin and DC-SIGN.^[6,168,214,215]^ In general, a larger subset of group 5 CTLD-containing proteins has been indicated to interact with highly sulfated oligosaccharides: NKG2 A & NKG2 C, CLEC-2, LOX-1 and CD94.^[38,214,255–257]^

Due to its involvement in chronic inflammation, NKG2D has sparked interest as a drug target.^[258]^ Two distinct scaffolds were found to inhibit protein-protein interactions of NKG2D *via* a proposed allosteric mechanism. Both ligands bind to the homodimer in a cryptic binding site in the interaction surface of the subunits (Figure 10b). The ligand expands a small pocket between the NKG2D domains and acts as a wedge between the monomers by replacing reciprocal hydrogen bonds between L148_A_ and K150_B_/L148_B_ and K150_A_ (Figure 10d). This way, the ligand disrupts the quaternary protein structure, distorts the protein-protein interaction interface, and abrogates binding of MHC-I-like protein ligands.^[131,135]^ The small-molecule ligands for NKG2D are a good demonstration of how allosteric and, in this case, cryptic sites can be used to bypass the usually low druggability of protein-protein interaction interfaces.^[259]^

NKG2D stands representative for group V CLRs that lack canonical Ca^2+^ binding capabilities and engage in protein-protein interaction.^[260]^ Systematic evaluation of CLRs (not only group V) has shown that most surfaces of the domain can be utilized for protein-protein interaction, hinting at the CTLD fold serving as a ‘universal binder’.^[107]^

### Concluding Remarks

For numerous CTLs, a single binding site is predominantly described. In the case of carbohydrate binding CTLs, this is referred to as the canonical CBS. Characterized by a Ca^2+^ -dependent anchoring interaction with a monosaccharide, residues proximate to the canonical CBS extend this site by forming additional interactions with substituents of larger glycoconjugates. However, variations exist where non-canonical and additional binding sites are utilized, with some proteins featuring two or more distinct sites. These non-canonical and secondary sites generally do not depend on Ca^2+^-coordination and can be located remote or proximal to the canonical CBS. They represent diverging features, broadening the repertoire of ligands interacting with CLRs.

**Figure 11:**
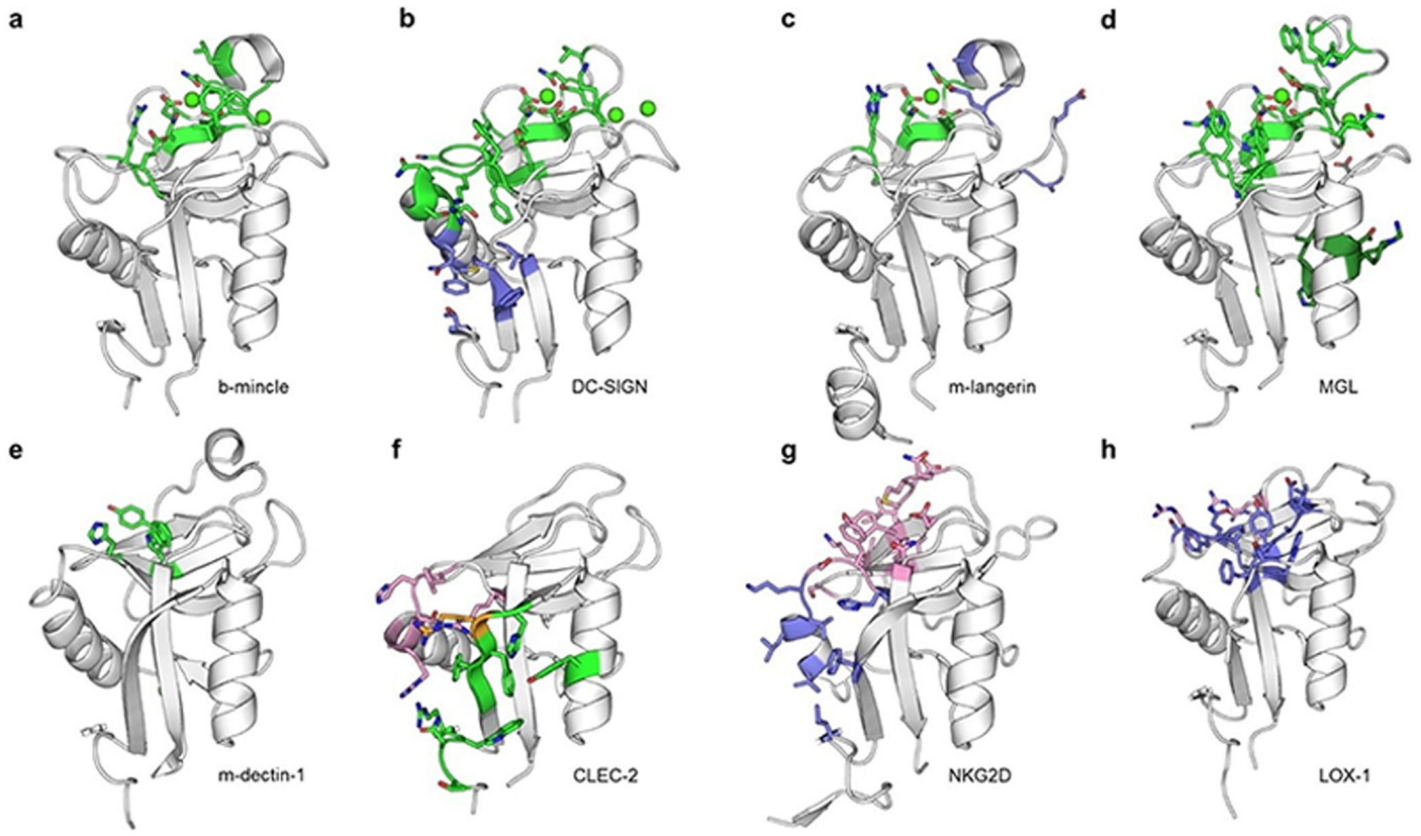
Summary of the protein X-ray crystallographic structures shown in this review illustrating the different binding sites utilized by the CTLD fold. The backbone of the CTLD is displayed in cartoon representation with Ca^2+^ ions depicted as green spheres; complexed ligands are hidden for clarity. Glycan-interaction sites are colored green, small molecule binding sites are colored blue, and protein-protein interaction sites are shown in pink. Side chains of amino acids highlighted by any color are depicted as sticks. CTLDs of group II CLRs are shown in the upper row (a-d), and CTLDs of group V CLRs are shown in the lower row (e-h): (a) (bovine) mincle (PDB ID: 4ZRV) shows canonical carbohydrate recognition; amino acids with 5 Å of bound glucose (not shown) are colored green.^[56]^ (b) DC-SIGN (PDB ID: 1 K9I) binds carbohydrates via the canonical CBS. For oligosaccharide ligands, the binding site is extended by a proximal binding pocket. Amino acids with 5 Å of bound GlcNAc_2_Man_3_ (not shown) are highlighted in green to illustrate this extended binding site.^[168]^ In addition, DC-SIGN binds a biphenyl mannoside via a Ca^2+^-independent binding site in the lower lobe of the CTLD mapped by ^1^H-^15^N HSQC NMR and shown in blue.^[13]^ Four additional binding sites for drug-like fragments have been experimentally verified (not shown).^[124]^ (c) (murine) langerin (PDB ID: 3P5D) binds carbohydrates in the canonical CBS, amino acids within 5 Å of glucose (not shown) are depicted in green. The binding site for thiazolopyrimidines in the loop region of the protein was mapped by solution paramagnetic relaxation enhancement in ^1^H-^15^N HSQC NMR and is shown in blue.^[132]^ The recognition of sulfated sugars via an extended binding site and a remote secondary binding site on human langerin are not shown.^[189,191]^ (d) MGL (PDB ID: 6PY1) binds carbohydrates in the canonical CBS; amino acids within 5 Å of N-acetylgalactosamine (not shown) are colored in green.^[207]^ A remote secondary site utilized for binding of *E. coli* R1 lipooligosaccharide (LOS) in the lower lobe of the CTLD was mapped by CSP in ^1^H-^15^N BEST-TROSY NMR and is shown in dark green.^[101]^ (e) (murine) dectin-1 (PDB ID: 2BPE) shows Ca^2+^-independent recognition of β-glucans. Essential amino acids forming the putative binding site in the upper lobe of the protein are colored green.^[99,100]^ (f) CLEC-2 (PDB ID: 3WSR) exhibits binding of a glycopeptide of PDPN via a remote binding site in the lower lobe of the CTLD. Amino acids making up the binding locus for the peptide part of the ligand (not shown) are colored pink, and amino acids making up the binding locus for the carbohydrate part of the ligand (not shown) are in green. R118 is part of both loci and is shown in orange.^[98]^ (g) NKG2D (PDB ID: 8EA6) has a binding site for a small-molecule ligand in the interaction interface of the physiologically functional homodimer (both small molecule and second monomer not shown). Amino acids within 5 Å of the small molecule are colored blue.^[135]^ The small molecule ligand prevents binding of the NKG2D homodimer to endogenous protein ligands containing an α1α2 platform domain. Amino acids within 5 Å of MICA in the complex with the NKG2D dimer (PDB ID: 1HYR; not shown) are highlighted in pink.^[105]^ (h) LOX-1 (PDB ID: 6TL9) is a functional homodimer (second monomer not shown) that recognizes protein ligands like oxLDL via the upper lobe of the domain. Amino acids of the so-called basic spine that mediates this interaction are shown in pink.^[40,41]^ Further, LOX-1 binds small-molecule ligand BI-0155 (not shown) in an additional binding site located in the upper lobe of the domain. Two BI-0155 molecules link two LOX-1 dimers, forming an inactive tetramer.^[89]^

An increasing amount of structural and biophysical data is now available and has led to the identification of binding sites spread across the entire CTLD fold, with instances where secondary binding sites in one protein may serve as the primary binding site in another (Figure 11). This observation underscores the evolutionary versatility and functional adaptability of the CTLD but also illustrates the phenomenon of either convergent or divergent evolution, leading to a diversification of binding sites within a conserved fold.

In a functional context, engagement of secondary sites enables unprecedented modes of modulating the biological responses triggered upon CLR engagement. Mechanisms ranging from chelation-derived avidity enhancement to allosteric activation (Example 1: DC-SIGN) or inhibition through changes in secondary (Example 2: langerin) and quaternary (Example 6: NKG2D) structure have been described for CLRs. In particular, endocytic Ca^2+^-dependent CLRs have been shown to exert a high degree of conformational plasticity, allowing for fine-tuning of ligand recognition and release at the CTLD, as well as larger pH-dependent rearrangements of the whole domain architecture. Previous studies have suggested evolutionary conservation and coevolution of allosteric networks across other protein families, pointing towards allostery to represent a common feature of the CTLD fold as well.^[17,261–263]^ Consequently, investigating the conservation of potentially allosteric secondary sites across the whole CTL family could shed light on how the CTLD has evolved alongside the different biological processes they are involved in. As for other protein families that have been previously deemed ‘hard-to-drug’, a detailed understanding of allosteric mechanisms and their physiological consequences could fuel the creation of rational concepts for the discovery of allosteric modulators interacting with secondary sites.

## Data Availability

Data sharing is not applicable to this article as no new data were created or analyzed in this study.
